# Severe Hypertriglyceridemia-Induced Pancreatitis Presenting as Diabetic Ketoacidosis in a Young Adult With Type 2 Diabetes Mellitus

**DOI:** 10.7759/cureus.84322

**Published:** 2025-05-18

**Authors:** Dhayananth Rattaipalivalasu Saravanan, Salil Avasthi, Chintan Desai, Maryam Saghir, Arzoo Khadka

**Affiliations:** 1 Internal Medicine, Mercy Health-St. Vincent Medical Center, Toledo, USA; 2 Pulmonary and Critical Care Medicine, Mercy Health-St. Vincent Medical Center, Toledo, USA; 3 Internal Medicine, Bon Secours Mercy Health, Cincinnati, USA

**Keywords:** acute pancreatitis, diabetic ketoacidosis (dka), high anion gap metabolic acidosis, hypertriglyceridemia induced pancreatitis, resistance to insulin, therapeutic plasmapheresis

## Abstract

Hypertriglyceridemia-induced pancreatitis (HTGP) is an uncommon but serious condition typically associated with markedly elevated triglyceride levels, often exceeding 1,000 mg/dL and more frequently seen above 2,000-3,000 mg/dL. Diabetic ketoacidosis (DKA), a common complication in patients with poorly controlled diabetes, may contribute to or be worsened by hypertriglyceridemia due to insulin deficiency and increased lipolysis. We report the case of a 20-year-old male with type 2 diabetes mellitus who presented with DKA and was subsequently diagnosed with HTGP. The patient experienced significant clinical improvement following urgent plasmapheresis, which resulted in a rapid decline in triglyceride levels. This case underscores the importance of considering hypertriglyceridemia as a potential underlying cause in patients with DKA and abdominal pain and highlights the therapeutic role of plasmapheresis in achieving metabolic stabilization.

## Introduction

Acute pancreatitis (AP) is a common inflammatory condition of the pancreas with multiple etiologies, including gallstones, alcohol use, medications, and metabolic disorders. Hypertriglyceridemia (HTG) is an infrequent cause of AP, accounting for up to 10% of cases [1]. Pathogenesis involves the hydrolysis of triglycerides by pancreatic lipase into free fatty acids, which exert a direct cytotoxic effect on pancreatic acinar cells. Severe HTG-often defined as serum triglyceride levels >1,000 mg/dL-can occur in the setting of insulin deficiency, most notably in diabetic ketoacidosis (DKA), due to enhanced lipolysis and hepatic very-low-density lipoprotein (VLDL) production [2].

Prompt identification of HTGP and rapid reduction of triglyceride levels are essential to prevent organ failure. Plasmapheresis has emerged as a therapeutic option for rapidly lowering serum triglycerides in cases resistant to conventional therapies [3,4].

## Case presentation

A 20-year-old male with obesity, type 2 diabetes mellitus (T2DM) on metformin and dapagliflozin, and occasional alcohol use presented with left-sided chest and flank pain, nausea, vomiting, and shortness of breath. He denied any change in bowel or bladder habits. Initial vital signs revealed hypertension (167/108 mmHg) and tachycardia (HR 128 bpm). He was afebrile and saturating well on room air.

**Figure 1:**
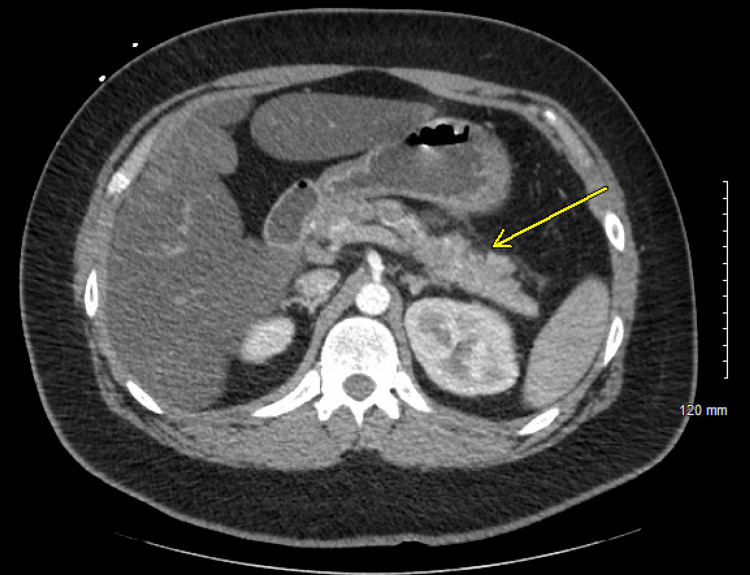
CT abdomen with contrast showing signs of acute pancreatitis

Initial labs, shown in Tables 1-3, were notable for leukocytosis, hyponatremia (Na 119 mmol/L), hyperglycemia (glucose 355 mg/dL), anion gap metabolic acidosis (anion gap 22), elevated beta-hydroxybutyrate, and elevated lipase (382 U/L). Urinalysis showed glycosuria, ketonuria, and proteinuria. Computed tomography (CT) imaging (Figure 1) revealed acute pancreatitis with no gallstones or biliary obstruction.

**Table 1:** Admission laboratory values BUN: Blood Urea Nitrogen; CO₂: Carbon Dioxide; HbA1c: Hemoglobin A1c

Parameter	Value	Reference Range	Interpretation
Sodium	119 mmol/L	135–144	Critically low
Potassium	4.0 mmol/L	3.7–5.3	Normal
Chloride	84 mmol/L	98–107	Low
CO₂ (Bicarbonate)	13 mmol/L	20–31	Low
Anion Gap	22 mmol/L	9–17	High
Glucose	355 mg/dL	70–99	High
Beta-hydroxybutyrate	9.76 mmol/L	<0.6	High
Lipase	382 U/L	13–60	High
HbA1c	11.5%	4.0–6.0%	High

**Table 2:** Lipid panel and inflammatory markers HDL: High-Density Lipoprotein, LDL: Low-Density Lipoprotein, CRP: C-Reactive Protein, Chol/HDL Ratio: Total Cholesterol to High-Density Lipoprotein Ratio

Parameter	Value	Reference Range	Interpretation
Triglycerides	3,070 mg/dL	<150	Critically high
Total Cholesterol	648 mg/dL	0–199	High
HDL Cholesterol	9 mg/dL	>40	Low
LDL, Direct	23 mg/dL	<100	Normal
Chol/HDL Ratio	72.0	<5	High
CRP	30.6 mg/L	0.0–5.0	High

**Table 3:** Arterial blood gas analysis pCO₂: Partial pressure of carbon dioxide, pO₂: Partial pressure of oxygen

Parameter	Value	Reference Range	Interpretation
Venous pH	7.037	7.320–7.430	Critically low
Venous pCO₂	15.7 mmHg	41.0–51.0 mmHg	Low
Venous pO₂	85.3 mmHg	30.0–50.0 mmHg	High (venous)
Venous Bicarbonate	4.2 mmol/L	22.0–29.0 mmol/L	Critically low
Base Excess	-23.9	0.0–2.0 mmol/L	High deficit

The patient was admitted to the ICU on hospital day 0 with a diagnosis of diabetic ketoacidosis (DKA) and acute pancreatitis. He was started on insulin infusion and intravenous fluids per standard DKA protocol. However, his DKA was unusually severe and prolonged, with persistent anion gap acidosis and critically low bicarbonate levels. Given the atypical severity and lack of response to standard therapy, a lipid panel was obtained on hospital day 0, revealing a triglyceride level of 3,070 mg/dL. Therapeutic plasmapheresis was performed on hospital day one, within 48 hours of admission. Following the procedure, there was a rapid and significant drop in triglyceride levels, which coincided with clinical stabilization. The trend in triglyceride levels during hospitalization is summarized in Table 4.

**Table 4:** Triglyceride trend during hospitalization

Hospital Day	Triglyceride Level (mg/dL)	Clinical Note
Day 0 (AM)	3,070	Initial lipid panel on admission
Day 0 (PM)	2,248	Pre-plasmapheresis
Day 1	481	Post-plasmapheresis
Day 2	323	Continued decline
Day 3	241	Continued decline

Therapeutic plasmapheresis was performed on hospital day one, within 48 hours of admission. Following the procedure, there was a rapid and significant drop in triglyceride levels, which coincided with clinical stabilization.

Given the severity of pancreatitis and persistent metabolic acidosis, it took nearly four days for the DKA to fully resolve, underscoring the profound systemic impact of hypertriglyceridemia in this case. Gemfibrozil was initiated on hospital day one, concurrent with plasmapheresis, to facilitate long-term triglyceride control. As the patient’s acid-base status improved and the anion gap closed, he was successfully transitioned to subcutaneous insulin and discharged in a stable condition with close outpatient follow-up.

## Discussion

This case exemplifies the pathophysiological connection between DKA and HTGP. Insulin deficiency in DKA stimulates hormone-sensitive lipase, promoting the release of free fatty acids and increased hepatic VLDL production, which can lead to severe hypertriglyceridemia [2].

HTGP should be suspected in patients with unexplained pancreatitis or DKA with abdominal pain and no obvious precipitating factor. Our patient’s triglyceride (TG) level of 3,070 mg/dL exceeded the threshold typically associated with pancreatitis and required urgent intervention. While insulin and hydration remain first-line therapy, plasmapheresis is recommended in cases with extremely elevated TG levels and ongoing systemic compromise [3,4]. It can rapidly reduce serum triglyceride concentrations and may decrease the inflammatory burden and risk of organ dysfunction.

## Conclusions

This case highlights the bidirectional relationship between diabetic ketoacidosis (DKA) and hypertriglyceridemia-induced pancreatitis (HTGP), particularly in young adults with poorly controlled type 2 diabetes. DKA can precipitate severe hypertriglyceridemia, leading to acute pancreatitis, a life-threatening complication if not promptly recognized. While insulin and fluids are first-line therapy, plasmapheresis serves as a valuable adjunct in cases with significant metabolic instability, enabling rapid clinical improvement.

Clinicians should suspect HTGP in DKA patients with abdominal pain, especially when typical causes like gallstones or alcohol are absent. Early lipid profiling and timely intervention, including consideration of extracorporeal therapies, are crucial. As obesity and diabetes rise among younger populations, such cases may become more frequent. This report underscores the need for standardized diagnostic criteria and evidence-based thresholds for plasmapheresis in HTGP.
